# Maturitas: Development and implementation of a longitudinal curriculum to foster Professional Identity Formation at the medical faculty of the University of Augsburg

**DOI:** 10.3205/zma001833

**Published:** 2026-03-23

**Authors:** Sabine Drossard, Sandra Schuh, Florian Gerheuser, Iris Warnken, Babette Schöningh, Anja Härtl

**Affiliations:** 1 University Hospital Würzburg, Department of General, Visceral, Transplant, Vascular and Pediatric Surgery, Würzburg, Germany; 2 University Hospital Augsburg, Department of Dermatology and Allergology, Augsburg, Germany; 3 University of Augsburg, Faculty of Medicine, Medical Didactics and Educational Research, DEMEDA, Augsburg, Germany; 4 University Hospital Augsburg, Department of Anaesthesiology and Intensive Care Medicine, Augsburg, Germany; 5 Praxis für Psychotherapie, Augsburg, Germany; 6 University Hospital Augsburg, Department of Hygiene and Environmental Medicine, Augsburg, Germany

**Keywords:** professional identity development, longitudinal curriculum, mentoring, curriculum development

## Abstract

**Background:**

The promotion of Professional Identity Formation (PIF) is gaining increasing international attention. At the newly established Medical Faculty of Augsburg, the longitudinal PIF curriculum *Maturitas* was developed based on the Canadian model at McGill University and implemented within the core curriculum beginning in the winter semester 2019/2020.

**Aim:**

This manuscript describes the development process during the first four semesters. The process can be divided into three phases, with a particular focus on the second phase. A central conceptual shift was to understand professional identity development as a continuous process from layperson to physician, highlighting the intermediate identity of “being a medical student” as a distinct developmental stage.

**Approach:**

Within *Maturitas*, both curricular teaching formats and a mentoring program were introduced and successfully established. The developmental process was accompanied by literature review, formative evaluation, and continuous feedback from students and faculty. Reflective analysis resulted in the identification of key enabling factors and challenges for the implementation and sustainability of PIF curricula.

**Results:**

Initial feedback indicates positive effects such as strengthened reflective capacity. However, challenges remain, including variable participation rates and the lack of systematic assessment of defined learning outcomes. Open questions include how the long-term effectiveness and sustainability of the curriculum can be demonstrated, how PIF-related content can be integrated across subjects, and how faculty-wide competence curricula can be implemented in a resource-efficient manner.

**Conclusion:**

The Augsburg experience illustrates that longitudinal PIF curricula can be successfully integrated into a core medical curriculum. At the same time, ongoing critical evaluation, iterative adaptation, and long-term assessment are required to meaningfully anchor professional identity formation alongside knowledge acquisition and clinical skills training.

## Abstract (de)

Die Förderung der professionellen Identitätsbildung (Professional Identity Formation, PIF) gewinnt international zunehmend an Bedeutung. An der neu gegründeten Medizinischen Fakultät Augsburg wurde das longitudinale PIF-Curriculum „Maturitas“ in Anlehnung an das kanadische Modell der McGill University entwickelt und seit dem Wintersemester 2019/2020 im Regelcurriculum implementiert.

Dieses Manuskript beschreibt den Entwicklungsprozess in den ersten vier Semestern, der sich in drei Phasen gliedern lässt und insbesondere in der zweiten Phase detailliert analysiert wird. Ein zentraler Aspekt war, die professionelle Identitätsentwicklung als kontinuierlichen Prozess vom Laien hin zur ärztlichen Rolle zu verstehen, wobei die Entwicklung einer Identität als Medizinstudierende:r als Übergangsstadium in den Fokus gerückt wurde.

Im Rahmen von Maturitas wurden sowohl Lehrveranstaltungen als auch ein Mentoring-Programm eingeführt und erfolgreich verstetigt. Der Entwicklungsprozess wurde durch Literaturrecherchen, Evaluationen und kontinuierliches Feedback begleitet. Die kritische Reflexion ermöglichte die Ableitung von Gelingensfaktoren und Stolpersteinen für die Implementierung und Verstetigung von PIF-Curricula.

Erste Rückmeldungen deuten auf förderliche Aspekte wie eine gestärkte Reflexionsfähigkeit hin, zeigen aber auch Herausforderungen wie schwankende Teilnahmequoten und die bislang fehlende systematische Überprüfung der definierten Lernziele. Offen bleibt, wie sich die Wirksamkeit und Nachhaltigkeit des Curriculums langfristig darstellen, wie PIF-Inhalte in andere Fächer integriert werden können und wie sich fachübergreifende Kompetenzcurricula ressourcenschonend implementieren lassen.

## 1. Introduction

Promoting Professional Identity Formation (PIF) has emerged as a central priority in undergraduate, postgraduate, and continuing medical education. The concept and definition of PIF have been discussed extensively in recent years. PIF is understood as an iterative process in which professional beliefs, values, and behaviors are constructed, deconstructed, and integrated into an evolving personal identity [1]. Role models play a key role in this process, as learners draw on them to internalize professional values, attitudes, and principles [2]. PIF is therefore closely linked to socialization processes and incorporates learners’ prior experiences [3].

In Germany, structured approaches to fostering PIF are still developing. The aim of PIF-related teaching is to support students in shaping their professional identity by systematically addressing the cognitive, affective, and practical dimensions of the physician’s role. Some faculties have implemented such teaching formats, extracurricular programs, or integrated curricular elements [4], [5]; however, a systematic overview is lacking. It is likely that PIF-related content is already being addressed implicitly or explicitly within various courses. The University of Jena introduced the longitudinal PIF curriculum LongProf in the winter semester 2021/2022 [6], [7], which is currently being piloted at Charité Berlin [https://allgemeinmedizin.charite.de/studium_lehre/longprof_charite/]. To the best of the authors’ knowledge, Augsburg was the first medical faculty in Germany to integrate a longitudinal PIF curriculum into the mandatory core curriculum for all students beginning in the winter semester 2019/2020.

This article describes the concept, development, implementation, and initial evaluation of a longitudinal curriculum to foster PIF among medical students at the newly founded Medical Faculty of Augsburg. We outline the multi-stage development process, discuss key challenges encountered during implementation, and highlight opportunities for further development and institutional integration.

## 2. Project description

**Figure 1:**
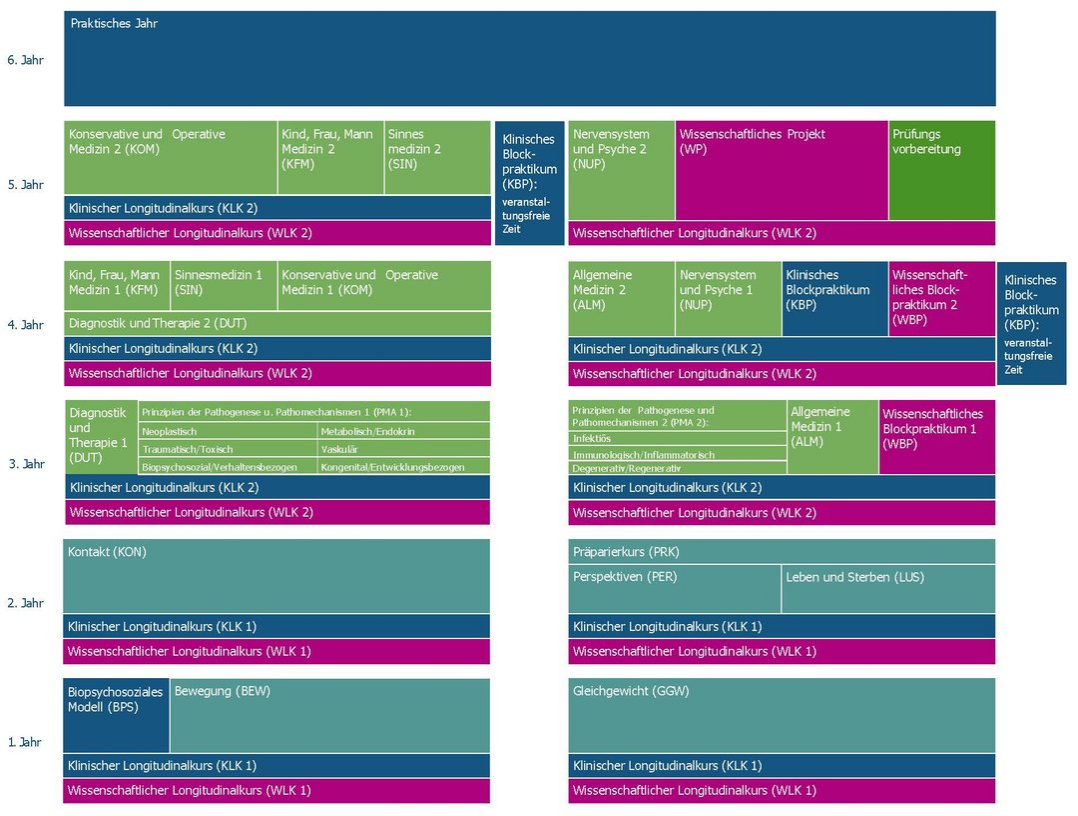
Structure of the medical curriculum at the medical faculty of the University of Augsburg (only in German) Source: https://www.uni-augsburg.de/de/fakultaet/med/studium/modellstudiengang-medizin/module/, accessed November 12, 2024

The medical faculty of Augsburg admitted its first cohort of 84 students in the winter semester 2019/2020. The curriculum was developed stepwise, with PIF established as a core thematic component from the outset (see figure 1 (Fig. 1)). The authors of this article were responsible for the development and implementation of the curriculum at different points in time and contributed with varying thematic emphases.

The development and implementation process from 2018 to 2022 can be divided into three phases. This manuscript focuses on the conceptualization and development of the PIF curriculum during the first four semesters of the new medical curriculum in Augsburg (winter semester 2019/2020 to summer semester 2021).

### 2.1. Phase 1: “Geneva declaration”

Following the founding of the medical faculty in 2016, curricular planning began parallel to organizational and staff development. A kick-off workshop with Yvonne Steinert (McGill University, Montréal) [8] in summer 2018 initiated the development of a PIF curriculum. A working group adapted the Professional Identity Formation Program established at McGill University [https://www.mcgill.ca/physicianship/] to the German context and the requirements of the National Competence-Based Learning Objectives Catalogue for Medicine (NKLM). In this model, the development of professional identity is understood as a socialization process in which learners internalize attributes, values, and norms of the medical profession. Central to this approach is the recognition that growing into the role of a physician requires not only the acquisition of knowledge (episteme, ἐπιστἠμη) and technical skills (techne, τέχνη), but also prudence and practical wisdom (phronesis, φρόνησις).

Based on an extensive literature review, the working group developed a conceptual framework including goals and key success factors. Initially integrated into the longitudinal clinical course, the PIF curriculum was later established as an independent program component within the overall curriculum.

The goals of the PIF curriculum were defined in 2018 during the project development phase and are summarized in table 1 (Tab. 1). The main thematic pillars were reflection, mentoring, and practice-oriented elements. Components of the Canadian program – such as the emphasis on the dual roles of *professional* and* healer* [9], and the focus on the *person who is ill* rather than *the illness* [10] – were not adopted. Instead, the articles of the Declaration of Geneva [11] were assigned to individual semesters to establish a longitudinal structural framework (see table 2 (Tab. 2)). During the initial module planning meetings, it was decided to introduce the PIF curriculum with a lecture at the beginning of the first semester, followed by two seminars and one small-group session each semester.

### 2.2. Phase 2: Maturitas

After the first semester, a new team took over coordination of the PIF curriculum. The program was institutionalized at the Institute of Medical Didactics and Educational Research and renamed *Maturitas* (Latin: *maturity*), a name suggested by the program’s patron, Prof. Wolfgang von Scheidt. At the same time, the new team faced the challenge of converting teaching activities to online formats due to the COVID-19 pandemic.

**Figure 2:**
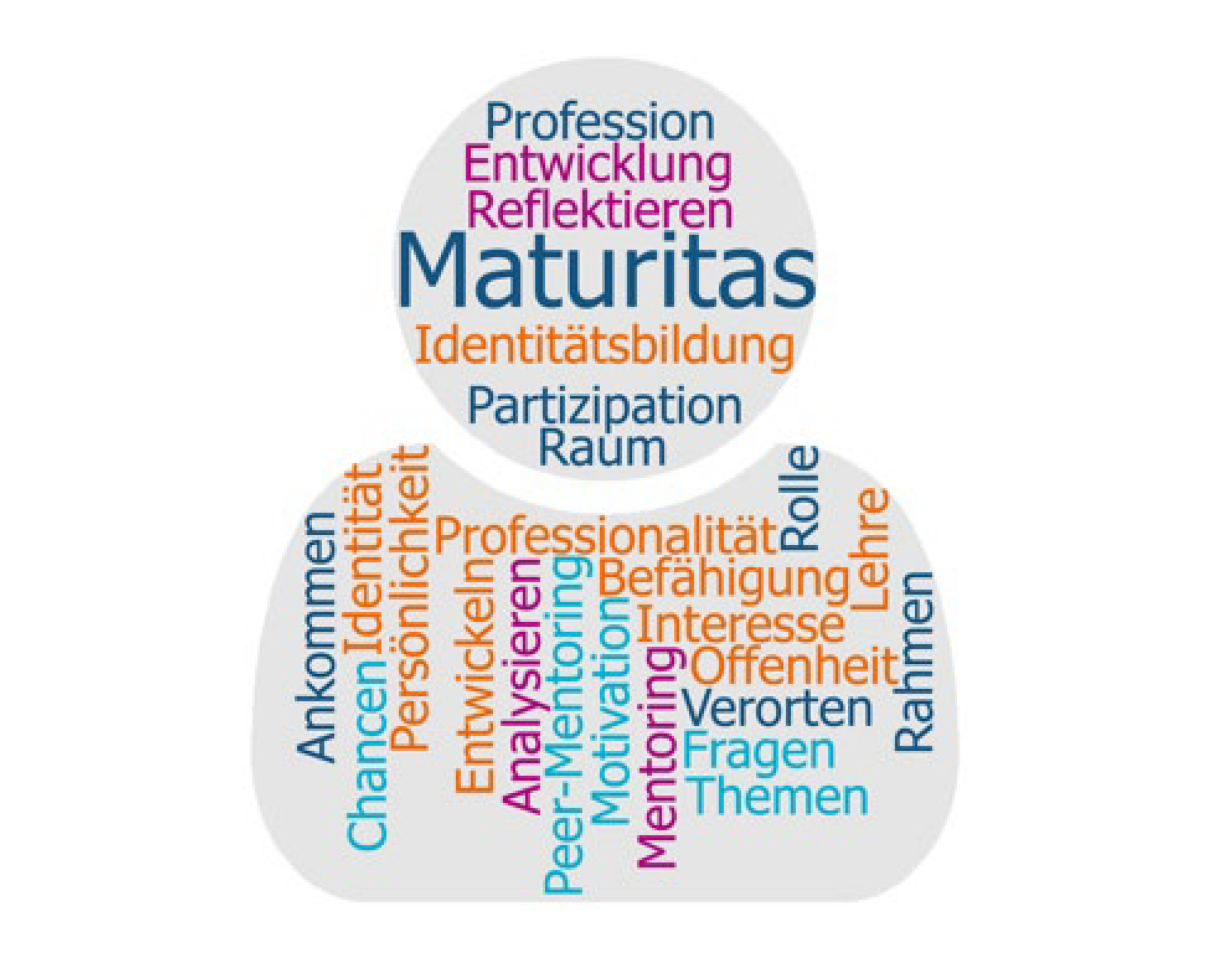
*Maturitas* visual identifier: Word cloud in the shape of a simplified human figure containing key terms from the *Maturitas* curriculum in Augsburg (only in German)

During this revision phase, a renewed needs assessment was conducted based on empirical findings and experiences of first-year students. The further development was guided by the recommendations of the GMA Committee on Communicative and Social Competencies (Undeloh Recommendations, 2015) [12]. *Maturitas* was presented on the Institutes website and a visual identity was created to ensure recognizability across all program activities (see figure 2 (Fig. 2)).

A browser-based project management tool was used to coordinate responsibilities, manage materials, and ensure transparent collaboration across university and hospital networks. *Maturitas* was regularly presented in curricular committees and a dedicated group email address was established to support communication.

#### 2.2.1. Thematic and content structure

A key publication by Cruess et al. (2015), which has strongly influenced the development of PIF in many medical curricula [13], conceptualizes PIF as a dynamic process shaped both by learners’ personal characteristics (“who they are”) and by the professional they aspire to become (“who they wish to become”) [14]. Learners respond differently to socialization influences, making PIF an inherently individual process [14]. The aim of *Maturitas* was therefore to support all students in their professional identity formation, taking into account their prior experiences. Since socialization also occurs implicitly within the “hidden curriculum”, and negative role models may reinforce undesirable attitudes and behaviors, the process was designed to be made explicit and guided.

Initially, the program focused on understanding students’ starting points and the socialization process itself. It was assumed that the transition from layperson to physician does not occur linearly but rather through intermediate stages. Before students identify with the physician role, they first develop an identity as *medical students* – a “semi-professional” role in which they are no longer laypersons, but also not yet physicians. Based on the premise that professional identity formation is never completed, the program’s goal shifted from *developing *identity to *enabling *and *supporting *identity formation.

*Maturitas* was structured into semester-specific themes aligned with the content of the clinical longitudinal course (Klinischer Longitudinalkurs, KLK) and the developmental stages of the medical program (see table 3 (Tab. 3)). 

To guide the design, the team drew on student feedback and the following guiding questions from the learner perspective:

- What concerns me in my current everyday reality?
- Which approaches and tools can help me address this?
- How can I apply these in concrete situations?
- How can I transfer what I have learned to future contexts?

The *Maturitas* team emphasized defining concrete and applicable learning objectives that would support students’ professional development. The clinical experience of the teaching staff (two residents and one physician with expertise in medical education) played an important role in shaping these practice-oriented approaches.

Three program components were defined:

1. *Maturitas* teaching sessions
2. *Maturitas* peer mentoring and mentoring
3. *Maturitas* extracurricular activities (not implemented due to the pandemic)

The didactic methods included guided perception, support, and reflection on personal development; orientation toward role models; and deliberate perspective-taking, including adopting the patient’s perspective. A practice-oriented approach was used to address students’ everyday concerns and accompany them through developmental challenges. For example, the seminar *“Please remove your shirt”* supported reflection on professional boundaries and interpersonal dynamics during peer physical examination. A cross-cutting seminar on *socialization* was also introduced. Topics were adapted based on student feedback. For instance, the session on *success and failure* was added at student request.

The program launched in summer semester 2020 with the *Maturitas* mentoring program, followed in winter semester 2020/2021 by an introductory lecture on the first day of the program and associated teaching sessions (see table 3 (Tab. 3)). In parallel, the *Maturitas* peer mentoring program was implemented, as previously described [15]. Online teaching included synchronous and asynchronous formats such as moderated discussion forums and written instructor feedback. Learning objectives addressing cognitive, affective, and practical dimensions were defined for each teaching session.

The program is allocated a fixed number of teaching hours within the curriculum and is counted as formal teaching workload according to university capacity regulations.

#### 2.2.2. Evaluation 

The curriculum was further developed on the basis of evaluation results and direct feedback from students. In the winter semester 2020/2021 and summer semester 2021, the *Maturitas* teaching sessions were evaluated using an online questionnaire assessing the relevance and organization of the topics. In addition, general questions about *Maturitas* were included. The questionnaire contained Likert-scale items as well as free-text fields. In the winter semester 2021/2022 and summer semester 2022, the *Maturitas *mentoring program was evaluated, supplemented by open-ended questions relating to the overall *Maturitas *curriculum. In addition, there was direct exchange with students.

Data analysis was carried out using EvaSys, and the free-text responses were categorized by the author team. Furthermore, attendance in the teaching sessions and participation in asynchronous online activities were evaluated, as there were no mandatory-attendance teaching sessions in Augsburg at that time. The extent of student participation was therefore also interpreted as an evaluation result.

### 2.3. Phase 3: Further development

From the winter semester 2022/2023 onward, the program was revised again, also due to personnel changes, and a new module team was established. Teaching sessions are now offered up to and including the 10^th^ semester.

*Maturitas* continues to be adapted in close coordination with students. For example, at the students’ request, the peer mentoring program was first extended from one to two semesters and, starting in the winter semester 2024/2025, to four semesters and renamed junior mentoring. The *Maturitas *mentoring program with clinically active mentors, which follows the peer mentoring, was renamed senior mentoring. While recruiting senior mentors was initially challenging, the program has since become well established, and clinicians now apply to act as mentors, resulting in a waiting list.

In the winter semester 2023/2024, as an additional component to provide collegial support to students in crisis situations, peer support was introduced in cooperation with PSU-Akut e. V. (Munich) [16]. This represents an adaptation of the “psychosocial support in health care” concept already established at the University Hospital Augsburg.

## 3. Results

The *Maturitas* teaching sessions, as well as the mentoring and peer mentoring programs, were successfully implemented and sustained. The transition of *Maturitas* to newly formed teams was facilitated by transparent communication and documentation of previous developmental steps, as well as the willingness of former team members to hand over responsibility while remaining available for questions. Anchoring *Maturitas* at the Institute of Medical Didactics and Educational Research also made visible that the program was supported at the faculty level. *Maturitas* is well known both within the faculty and at the university hospital and is valued by students and teaching staff.

Participation in the *Maturitas *teaching sessions varied. Attendance was particularly low during examination phases. In peer mentoring, many groups met more frequently than scheduled, whereas some senior mentoring groups only met once per semester, for example due to difficulties in finding suitable meeting times.

At the beginning of their studies, students found it difficult to identify with the physician role. This was evident, for example, in the seminar on dealing with errors, in which students expressed strong empathy for affected patients, while initially finding it difficult to adopt the perspective of the physicians involved in these cases.

Overall, students in the second semester evaluated the program more positively than students in the fourth semester. In the evaluation of the winter semester 2020/2021, 54.5% of students in the third semester stated that *Maturitas* was helpful for their personal development, and 36.4% considered the program helpful for their professional development (see table 4 (Tab. 4)). In the free-text responses, the added value of the sessions was frequently emphasized, even if some students only recognized this after the semester had ended (see table 5 (Tab. 5)). The mentoring program, as a component of *Maturitas*, was also perceived very positively. Teaching staff and mentors often expressed that they would have appreciated such a program during their own studies. The formal recognition of teaching hours was perceived by teaching staff as supportive, as it provided planning certainty and signaled appreciation of teaching activity.

The free-text responses showed that the defined goals of *Maturitas* were recognized by students (see table 5 (Tab. 5) and table 6 (Tab. 6)). The feedback from participating students was consistently positive, both during teaching sessions and in informal conversations and mentoring settings, even though it was repeatedly noted that participation in sessions declined during examination periods, as students prioritized exam-relevant courses. The asynchronous online format allowed students to engage with the content outside examination periods. The tools and prompts for reflection were perceived as helpful.

For the mentoring program, mandatory participation was tested for a limited period. However, as disinterested students did not attend the meetings and this led to frustration on all sides, the system was changed back to voluntary participation the following year. Both mentors and mentees clearly rejected mandatory participation, as mentoring is based on voluntariness, trust, and openness. The presence of students who did not wish to participate would have significantly impaired group dynamics and thus the effectiveness of the format. Similarly, attendance in *Maturitas *teaching sessions showed that small-group dynamics and learning atmosphere benefited from voluntary participation.

Both teaching staff and students appreciated the exchange and collaborative development of the program. At the students’ request, for example, the session on exam stress was supplemented with a seminar on learning strategies. Initial skepticism among teaching staff, especially regarding limited resources, decreased over time due to positive feedback.

## 4. Discussion

The conception and implementation of a longitudinal curriculum for professional identity formation in Augsburg was successfully achieved, although the development process was not linear. *Maturitas* is now firmly anchored within the Augsburg curriculum and well established among students and teaching staff. Feedback from students and the broad recognition of the program across the faculty indicate both positive effects and challenges. The evaluation results should be interpreted with caution due to the low response rates; they are to be understood as supplementary assessments from the student perspective rather than as representative findings. The somewhat lower acceptance in later semesters may have several causes, for example a shift in priorities among students or an increasing workload due to examinations.

The fixed allocation of teaching hours made it possible to integrate content that is otherwise more commonly found in elective offerings, but that is nevertheless important for study success, for the transition into professional practice, and for later clinical work. A central aspect of *Maturitas* is the stepwise alignment of the content: initially toward professional identity formation in the role of the medical student, and increasingly toward the physician role as the course of study progresses. Student feedback shows that focusing on concrete topics taken from students’ everyday experience facilitated the engagement with PIF and made the relevance of the content more apparent. This focus may therefore be understood as a key element contributing to the success of the PIF curriculum in Augsburg, as students were able to identify with the issues addressed and perceived the program as meaningful and relevant to their own development.

The learning objectives of the curriculum (see table 1 (Tab. 1)) have not yet been systematically evaluated. Initial indications can be seen, for example, in the mentoring program (reflective capacity) and in the frequent use of psychosocial support structures (self-care). Other effects, particularly those relating to the physician role, will only become apparent as graduates transition into professional practice and thus will only be measurable in longer-term evaluations.

Key enabling factors included a strong personal commitment of the team members throughout the various phases of development, enthusiasm of the teaching staff, and the opportunity to incorporate PIF into the curriculum from the beginning. Challenges also emerged, for example the fact that students tended to prioritize courses perceived as directly examination-relevant, especially toward the end of the semester – a phenomenon described at other sites as well [7]. 

Given limited teaching resources and the substantial developmental effort required at a newly established faculty, we also encountered the argument that “mandatory” subjects (medical content) had to take precedence over “optional” elements (such as PIF). Accordingly, the authors consider it essential to clarify from the outset whether a PIF program is regarded as a sufficiently high priority to justify the associated effort for both faculty and students. Central to this discussion is the question of whether and how learning progress in the sense of professional identity formation can and should be assessed. Internationally, different approaches exist, often focusing on guided reflection, for example through portfolios [4], [14]. The Augsburg team has so far deliberately chosen a voluntary model that relies on students’ intrinsic motivation. Our experience suggests that the quality of the program and positive peer recommendation represent sustainable factors in promoting participation.

Based on our experiences, student feedback in the context of participatory development, and discussions among the module coordinators, the following aspects were identified as particularly supportive for successful implementation of the PIF curriculum:

- explicit commitment of the faculty, particularly the dean’s office, to the PIF model based on an agreed definition
- formation of an interprofessional team including clinically experienced physicians
- early involvement of students
- ongoing exchange with teaching staff and curriculum coordinators
- clear communication within the faculty and university hospital
- early selection and training of appropriate teaching staff (role models)
- curricular integration on equal footing with other subjects (scope, attendance, assessment)
- continuous evaluation and willingness to revise and further develop the curriculum

During the second development phase, further factors supporting implementation became evident. Persuasion regarding a relatively unfamiliar topic was often successful only once concrete outcomes and examples were available. A pilot phase can therefore be considered valuable as a basis for further discussion. In addition, the choice of a poignant program name proved beneficial: *Maturitas* functioned as a communicative anchor, making both the program and the concept of PIF visible across the faculty.

The Undeloh recommendations of the GMA Committee on Communicative and Social Competencies (2015) [12] provide a helpful overview of key conditions for successful curriculum development. Many of these were also identified in our own reflection. *Student involvement* from the first semester onward contributed substantially to program acceptance and further development. *Attending to available resources* was only partially possible at the outset but improved over time. The *explicit institutional mandate* from the faculty came relatively late, which led to frustration for the first project group due to insufficient resources. *Needs assessment at individual, organizational, and contextual levels* was part of the process from the beginning but had to be revised several times due to changing targets. *Defining competencies* proved essential for continued development. *Methodological considerations* became particularly relevant during the COVID-19 pandemic, when online implementation also yielded unanticipated advantages, such as asynchronous usage, new forms of feedback, and expanded communication via forum and reflection tasks.

*Implementation* was coordinated using an online project management tool, enabling location-independent, transparent, and structured planning with clear responsibilities. Planning *Maturitas* along the semester progression and involving active clinicians ensured a link to clinical practice. A senior clinician acting as program sponsor supported program acceptance. *Evaluation* criteria and accompanying assessment were defined early. A key focus was to strengthen program acceptance and to demonstrate relevance to students – feedback indicates that this was largely achieved.

Stable integration into curricular planning through designated teaching hours, institutional anchoring at the institute of medical didactics, structured coordination, and transparent documentation of program development support *long-term sustainability. Maturitas* has been presented regularly in faculty committees from the outset and continues to be iteratively refined.

Although PIF-related content is implicitly present in various teaching contexts, it is not yet widely recognized as part of *Maturitas*. Following successful implementation, the next step is to integrate *Maturitas* explicitly into other subjects and teaching sessions and to further embed aspects that have so far received less emphasis.

## 5. Conclusion

With *Maturitas*, a longitudinal curriculum to foster professional identity formation was integrated into the core medical curriculum in Augsburg for the first time. A central element is the gradual alignment of the content, initially focusing on identity development in the role of the medical student and, as the course of study progresses, increasingly on the physician role. The implementation took place within an iterative process in which different project teams were involved, each contributing distinct focal points. The program is now firmly embedded in the curriculum and continues to be further developed. The stable integration into existing structures, the testing of new formats in pilot phases, and a participatory approach with continuous adaptation to the needs of students proved to be important factors for ensuring student acceptance and teaching staff motivation.

As the achievement of PIF-related learning objectives is difficult to measure and effects often become evident only later, studying long-term impact remains challenging. Initial feedback indicates positive effects, while also pointing to challenges such as fluctuating participation rates in voluntary components. In addition, the defined learning objectives have not yet been systematically evaluated; some effects will only become apparent when graduates enter clinical practice. For further development, it will therefore be necessary to create suitable evaluation instruments that can capture both short-term and long-term effects on professional identity formation.

The Augsburg experience demonstrates that the introduction of a longitudinal PIF curriculum is feasible, but requires continuous critical monitoring, iterative refinement, and long-term evaluation in order to establish professional identity formation as a third pillar of medical education alongside knowledge and skills acquisition.

## Acknowledgements

We would like to thank the medical faculty of the University of Augsburg/DEMEDA for their support in the development and implementation of the program, in particular the Dean of Studies Prof. Thomas Rotthoff and Dean Prof. Martina Kadmon. We especially thank the Augsburg medical students for their engagement, trust, and constructive feedback in the development of *Maturitas*. Our sincere thanks also go to all teaching staff who actively supported the project and/or served as mentors, as well as to Prof. von Scheidt for his patronage and for suggesting the name of our PIF curriculum.

## Authors’ ORCIDs

- Sabine Drossard: [0000-0002-3442-4851]
- Sandra Schuh: [0000-0002-1470-7619]
- Florian Gerheuser: []0009-0000-2680-9519
- Iris Warnken: [0009-0000-8497-2541]
- Babette Schöningh: [0009-0001-2309-106X]
- Anja Härtl: [0009-0008-0818-6213]

## Competing interests

The authors declare that they have no competing interests. 

## Figures and Tables

**Table 1:**
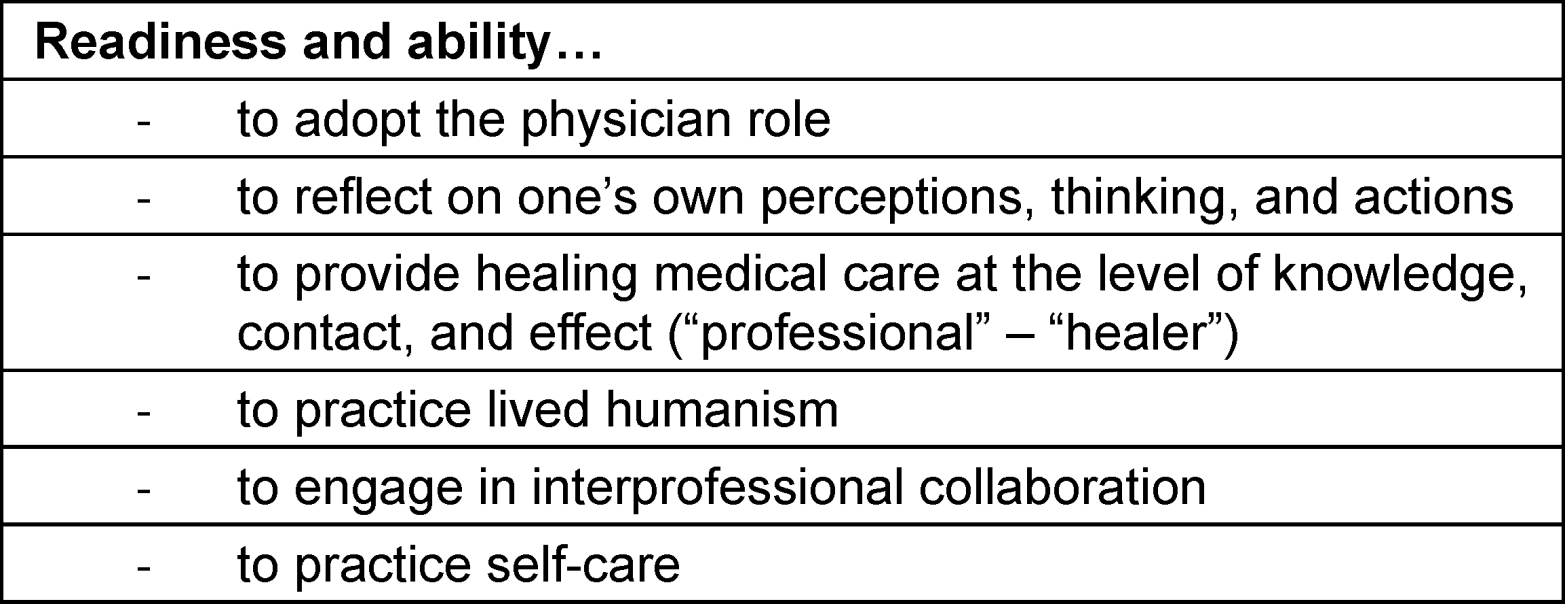
Goals of the Augsburg PIF program

**Table 2:**
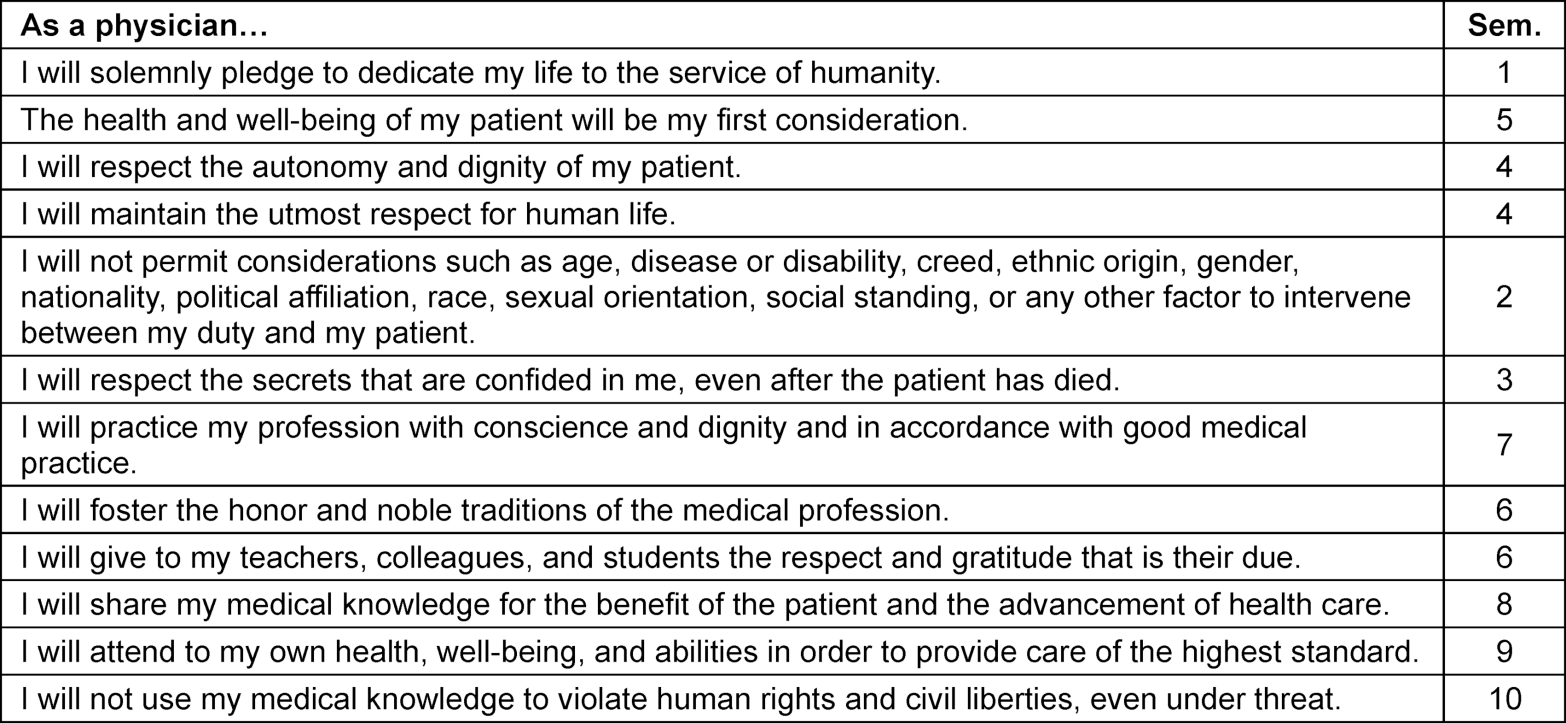
Assignment of articles of the declaration of Geneva to semesters (original planning 2019)

**Table 3:**
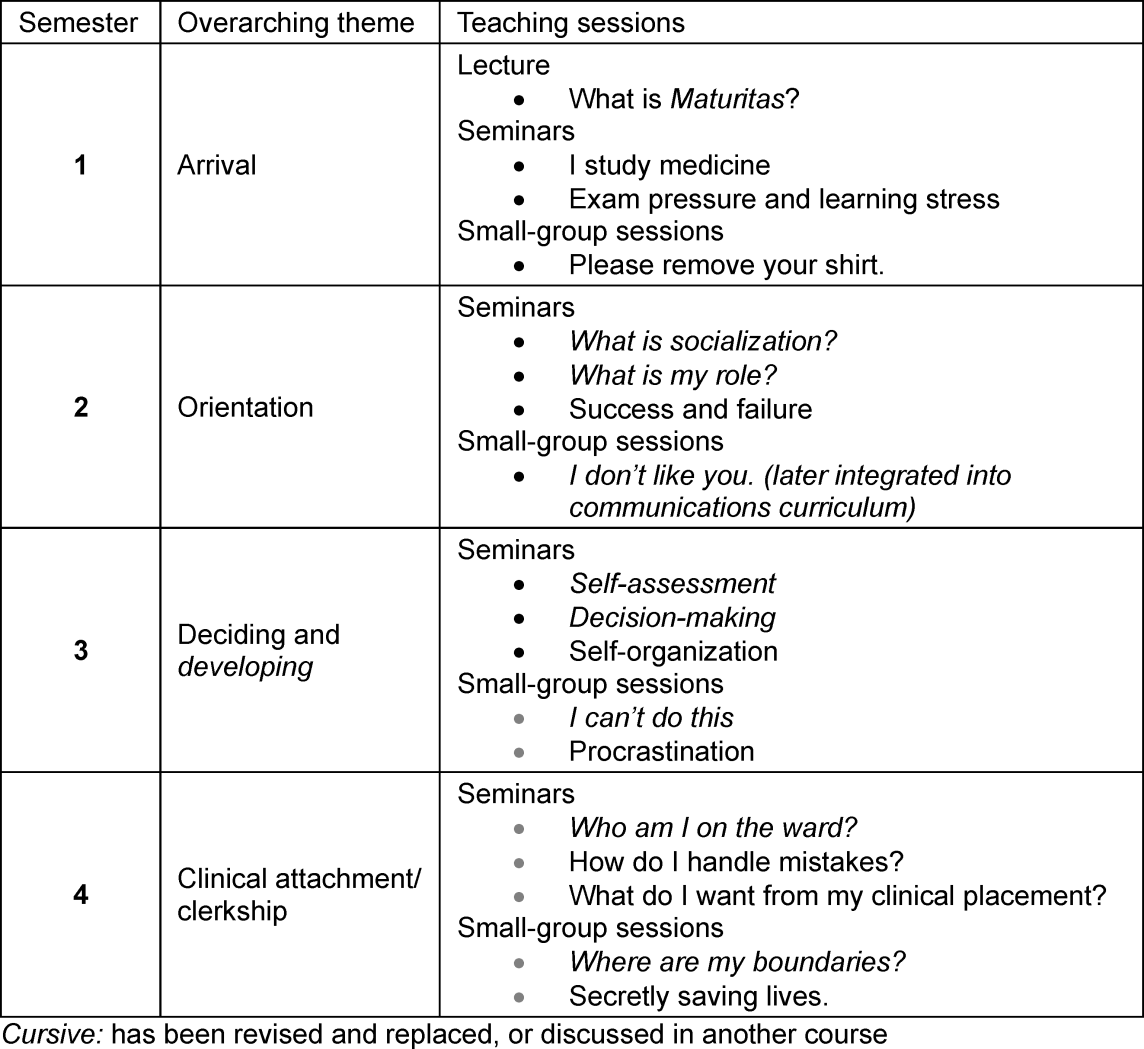
Overview of *Maturitas *teaching sessions

**Table 4:**
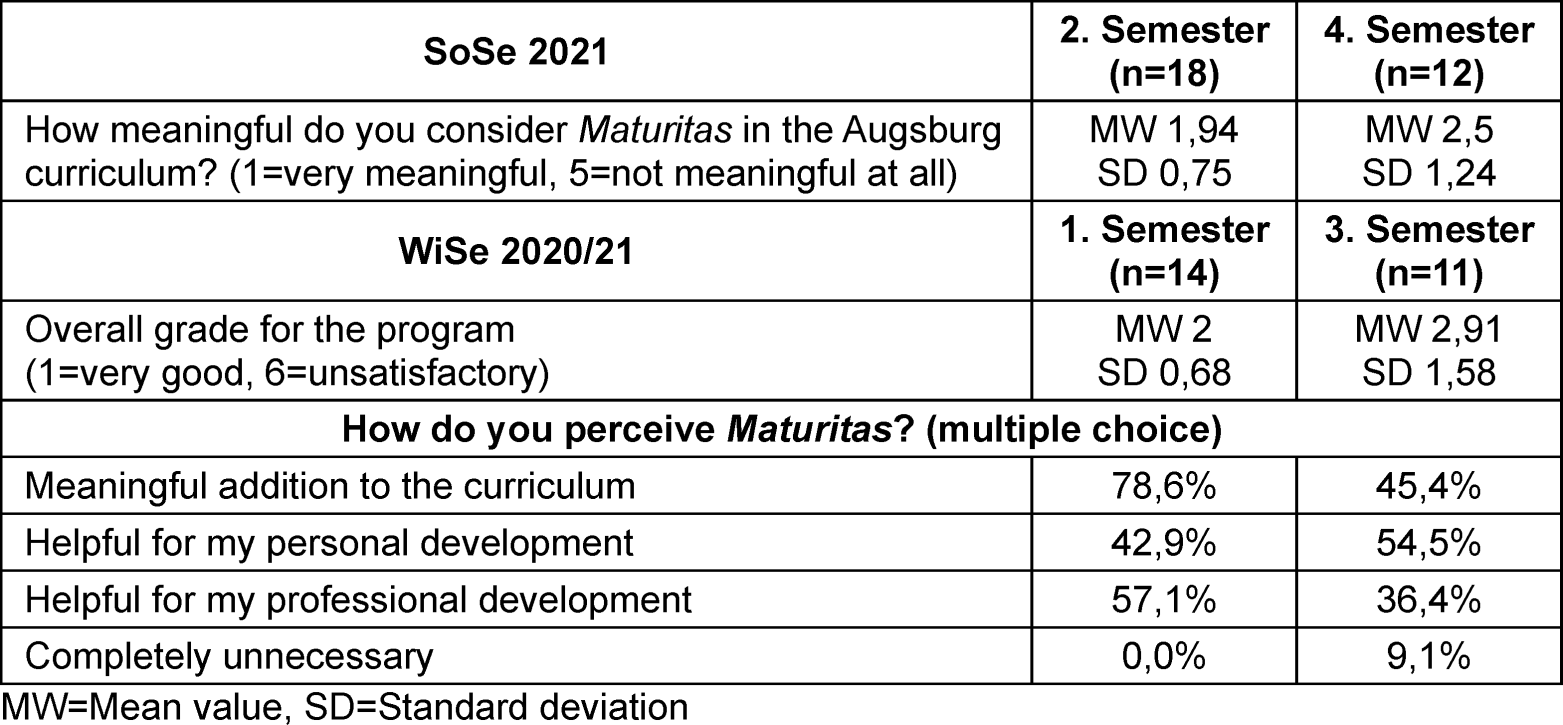
Evaluation results of the program (phase 2, year 1)

**Table 5:**
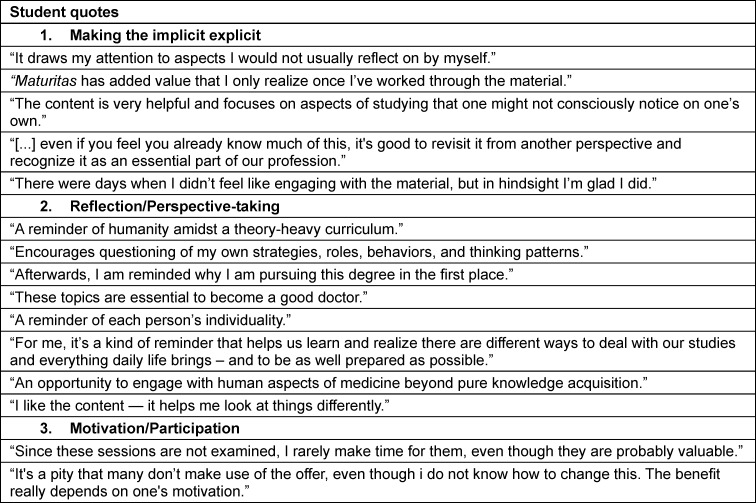
Selected free-text comments from the evaluation of *Maturitas* teaching sessions clustered by themes (WiSe 2020/21 and SoSe 2021)

**Table 6:**
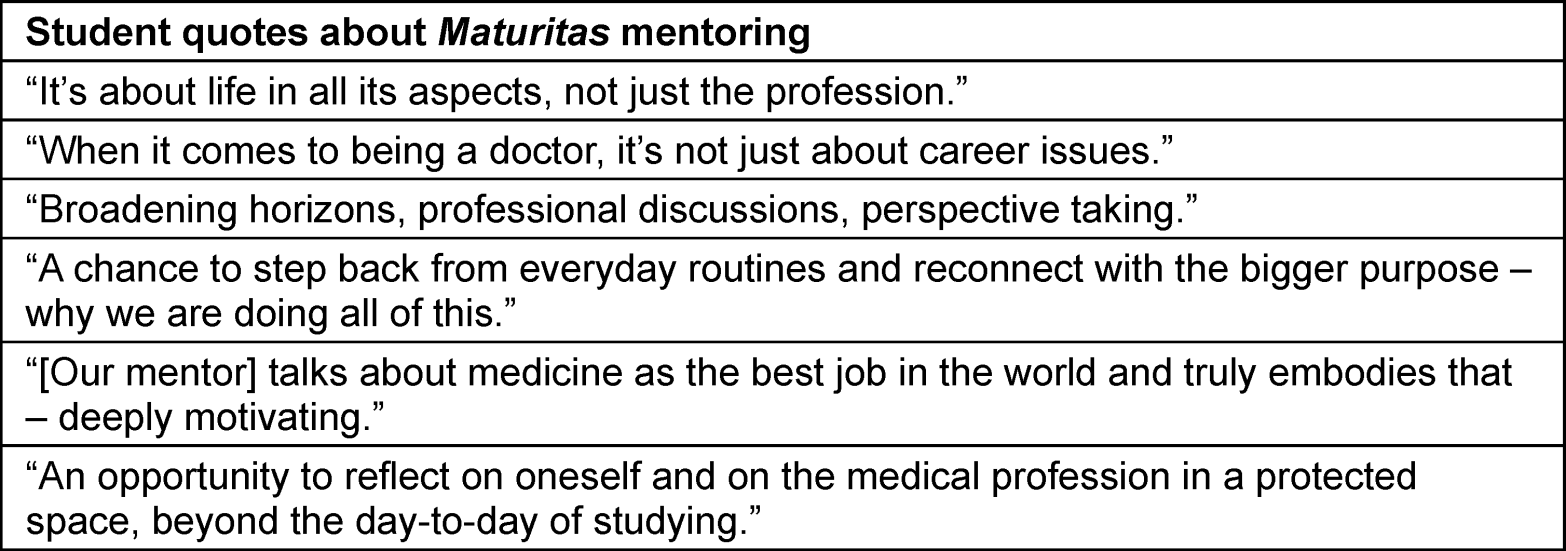
Selected free-text comments on *Maturitas* mentoring (WiSe 2020/21-SoSe 2022)
